# Mental Load and Fatigue Assessment Instruments: A Systematic Review

**DOI:** 10.3390/ijerph19010419

**Published:** 2021-12-31

**Authors:** Jesús Díaz-García, Inmaculada González-Ponce, José Carlos Ponce-Bordón, Miguel Ángel López-Gajardo, Iván Ramírez-Bravo, Ana Rubio-Morales, Tomás García-Calvo

**Affiliations:** 1 Faculty of Sport Sciences, University of Extremadura, 10003 Cáceres, Spain; jdiaz@unex.es (J.D.-G.); jponcebo@gmail.com (J.C.P.-B.); malopezgajardo@unex.es (M.Á.L.-G.); ivannramirezb@gmail.com (I.R.-B.); anarubmor94@gmail.com (A.R.-M.); tgarciac@unex.es (T.G.-C.); 2 Faculty of Education, University of Extremadura, 06006 Badajoz, Spain

**Keywords:** cognitive fatigue, mental health, assessment procedure, measurement, validity

## Abstract

Mental load and fatigue are important causes of performance decreases and accidents in different activities. However, a robust systematic review, detailing the instruments used to quantify them, is currently lacking. The purpose of this study was to summarize and classify by derivations the validated instruments used to quantify mental load and fatigue. The most representative electronic databases in the scope of this review, PubMed, WOS, Scopus, SPORTDiscus, and PsycINFO (until September 2020) were searched for studies that included instruments to analyze mental load and fatigue. The quality of the selected studies was scored using a quality assessment checklist. A total of 40 papers were included. Most of the papers used subjective scales (75%) to quantify mental load and fatigue, with a small presence of behavioral (*n* = 5) and objective techniques (*n* = 5). Less is known about the analysis of mental load and fatigue using a combination of derivations. Despite the high cost and complexity of objective techniques, research that applies these measures is important for further analysis of brain processes in mental load and fatigue. The design of a battery of tests that include the three types of derivations also seems necessary.

## 1. Introduction

Fatigue can be caused by excessive mental and/or physical demands, but the analysis of fatigue has focused on physical aspects [1]. Physical fatigue causes impairments in the traditional physiological variables (i.e., heartrate, blood lactate, or oxygen uptake). Contrary to physical fatigue, mental fatigue is not associated with these impairments, although the specific role of the brain has been demonstrated in mental fatigue [2]. Mental fatigue is apparently caused by excessive mental demands [3], and some authors have used the term “mental load” to refer to it [4]. Mental load and fatigue have been widely associated with specific performance decreases or an increase in the risk of accidents [1,5]. Although the impairments caused by mental aspects reveal the importance of quantifying these variables, a robust systematic review of the available instruments used to quantify mental load and fatigue is currently lacking. This information may enhance the importance of controlling these variables and facilitate experts’ choices of the most adequate instrument according to their needs.

Mental fatigue represents a psychobiological state with subjective (e.g., an increase in feelings of tiredness), behavioral (e.g., motivation decrease or reaction time increase), and physiological (e.g., alterations in the electroencephalogram signal) derivations in humans [6]. This psychobiological state is caused by brain-demanding tasks (i.e., mental load), with a relevant role of emotional (e.g., anxiety or stress) and cognitive (e.g., working memory or cognitive flexibility) aspects [6]. This should be considered in the analysis of mental load and fatigue, although most of the studies performed have used the cognitive aspects of mental fatigue [6]. For example, the case of Simon Biles or the different episodes observed during the COVID-19 are examples of how emotional aspects may impair health and performance.

Specifically, it has been observed that mental load and fatigue may impair human physical performance [6]. Some authors have stated that this phenomenon occurs through the increase in the subjective Ratio of Perceived Exertion, whereas other performance indicators, such as accuracy, tactical decisions, or reaction time, may be impaired by excessive accumulation of mental fatigue [1]. These impairments have been observed in different contexts such as medical surgery, construction work, or athletic settings [1]. Although the accumulation of extracellular adenosine or impairments in cognitive functions are possible explanations of this phenomenon, more studies are necessary to clarify the causes underlying these impairments.

However, it is difficult to analyze the causes and consequences of mental fatigue. Several covariables influence the mental fatigue induced by task performance, such as task difficulty, engagement, duration, or enjoyment/aversion [7]. In addition, a large number of individual differences could explain why the same task does not induce the same level of mental fatigue in different subjects or why mental fatigue manifests with different derivations (i.e., subjective, behavioral, or physiological) among participants [8]. Van Cutsem and Marcora [7] strongly recommend the use of a combination of several derivations (i.e., subjective, behavioral, and physiological) of mental fatigue as the best approach to identify its presence. Changes in all three areas do not necessarily appear in mentally fatiguing conditions, and they could depend on the subjects’ individual characteristics. For example, cognitive performance does not necessarily decline in presence of mental fatigue due to the effect of the compensatory effort system [6,7,8,9]. Therefore, the use of different measures of mental fatigue may identify the causes of mental fatigue or explain why mental fatigue impairs performance.

Despite these recommendations, few existing procedures allow experts to assess mental load and fatigue, making them difficult to control [3]. On the one hand, different instruments have been used for this purpose indirectly, subjectively, and behaviorally. For mental load, we find the (i) NASA Task Load Index [10], (ii) the Subjective Mental Workload Scale (SCAM) developed by Ceballos-Vásquez et al. [11], or (iii) the “StuMMBE-Q” [12], among others. For mental fatigue, the subjectively reported Visual Analogue Scale (VAS) has been the most used instrument. Despite the high reliability and validity of these instruments, information about brain processes is lacking. On the other hand, objective instruments have also been used for this purpose. Pupil dilation [13], eye tracking [10], and different electrophysiological indicators such as electroencephalography (EEG; [14]) or brain functional connectivity patterns [15] have been recommended by authors to quantify mental load and fatigue. Although these measures are necessary to increase the quality of the data of mental fatigue, the high cost and the low ecological validity of the data extracted (e.g., EEG requires a sedentary activity to perform the measures; therefore, in a sport-specific context, experts cannot quantify the mental activity) of many of these instruments makes their use difficult.

### The Present Study

Consequently, although the importance of mental variables in daily activities, work, or sports has increased because of the negative consequences of mental load and fatigue, it is difficult to choose a valid instrument to assess mental load and fatigue considering the different derivations caused by mental fatigue. Therefore, the first research question of this study is: What instruments exist to quantify mental load and mental fatigue? Consequently, the main purpose of this study was to summarize the instruments used to quantify mental load and fatigue. The second research question is: What instruments are more adequate to quantify each specific derivation of mental fatigue? Hence, we have also classified them by the type of derivation quantified to allow experts to choose the most adequate single instrument or battery of instruments, following the recommendations of using a combination of instruments for different derivations.

## 2. Materials and Methods

With this systematic review, we methodologically and comprehensively searched, appraised, and synthesized research evidence [16] for studies, aiming to identify the instruments used to quantify mental load and fatigue. This research was developed following the Preferred Report Elements for systematic reviews and meta-analyses (PRISMA) recommendations for systematic reviews [17] and the elements chosen for review [18]. Furthermore, this review was preregistered using the international prospective register of Systematic Reviews and Meta-analysis (PROSPERO [19,20]; registration: CRD42020167775).

### 2.1. Eligibility Criteria and Search Strategy

We followed the systematic review procedure suggested by Grant and Booth [16]. We included original empirical research papers published each year until September 2020 (i.e., we did not specify the start of the year, including all articles published until September 2020). Papers selected for analysis were found through searches of the most representative electronic databases for the scope of this review: PsycINFO, PubMed, Scopus, SPORTDiscus, and Web of Science. To identify the studies that used instruments to analyze mental load and mental fatigue, the authors used broad inclusion criteria, and all relevant research was included in the present study [21]. The following search terms were used to explore electronic journals: (i) mental load or mental fatigue; (ii) assessment OR measurement OR instrument; and (iii) validation. In each of the databases, the advanced search option was used to obtain the best combination and to access all possible research within our study framework. For example, in the Web of Science database, the following search was performed: TS = (“mental load” OR “mental fatigue”) AND TS = (“assessment” OR “measurement” OR “instrument”) AND TS = (“validation”). In addition to the search carried out in the databases, we performed a manual search to identify additional works to include in the study.

Before beginning the investigation, the inclusion and exclusion criteria were established to correctly define the objectives of this systematic review. Considering the search terms, we decided to include all the available works in each database, including all the languages present in each investigation. In addition, all articles published at any time before September 2020 were included. Another inclusion criterion was that all documents were original, with the full text available for analysis [22]. Articles with some measure or validity instrument on mental load or mental fatigue were also included.

### 2.2. Study Selection and Data Collection Process

**Figure 1:**
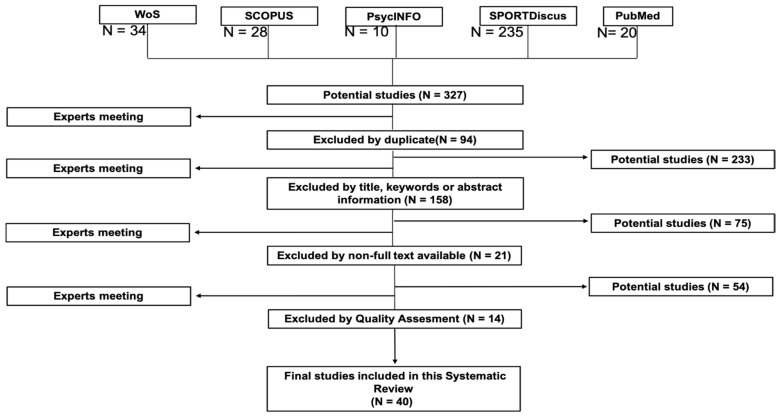
Process followed for the systematic review to classify by areas the validated instruments used to quantify mental load and fatigue.

Within the screening system, after reading the title and summary, the full text of the articles that were considered suitable for the review was selected to be evaluated and introduced into the study. The PRISMA flowchart (Figure 1) represents the filtering system for the final collection of the selected sample to complete the preparation of the current review. An expert meeting was held at each of the filters to determine the inclusion or exclusion of the different works previously analyzed. Figure 1 also shows the number of documents included and excluded in each of the phases of the screening process.

At the end of the search in the different databases, a total of 327 potential studies was obtained, of which 94 were eliminated for duplication and 158 due to the topic. Subsequently, 21 studies were eliminated for lack of the full text, and 14 because they did not meet the established quality criteria. After this selection process, a total of 40 articles was obtained.

All these steps were performed independently by two researchers following the same criteria. Kappa statistic (*k*) was employed to test the percentage of interrater agreement, indicating strong agreement between the two raters (*k* = 0.85, [23,24]). Discrepancies were discussed with a third reviewer until 100% consensus was reached.

### 2.3. Data Synthesis

Once the definitive studies were selected, a synthesis of the information and the most important characteristics of each article were extracted. Data relating to the instrument used, authors and year of publication, sample characteristics (i.e., number of volunteers, sex, age), and other instruments used for comparison were extracted. The results and conclusions to analyze the validity and reliability were collected. Thus, the studies were overviewed and compared, allowing us to evaluate the current state of research on mental load and mental fatigue assessment, which was divided into different sections. Due to the diversity of derivations through which mental load or mental fatigue was assessed, each document was assigned to one of the three subsequently established categories: (i) mental load and fatigue assessment instruments for subjective derivations; (ii) mental load and fatigue assessment instruments for behavioral derivations; (iii) mental load and fatigue assessment instruments for physiological derivations.

### 2.4. Quality Assessment

The quality of all studies was evaluated using the quantitative assessment tool ‘QualSyst’ [25]. This validated checklist consists of 14 sections, each assessing a different measurement property (see Table 1). Each item within a section is scored on a three-point scale depending on the degree to which the specific criteria were met (yes = 2, partial = 1, no = 0). A score of >0.75 indicated strong quality, a score between 0.75 and 0.50 indicated moderate quality, and a score <0.50 indicated weak quality. The term “NA” was used for those items without a particular study design, which were excluded from the calculation of the summary score. This process was carried out by two reviewers (M.A.L.G. and J.C.P.B.), and discrepancies were discussed with a third reviewer (J.D.G.) until 100% consensus was reached. Likewise, the Kappa statistic (k) was employed to test the percentage of interrater reliability [26]. These steps were performed by two reviewers. The agreement between researchers reflected in the kappa coefficient (κ = 0.84, *k* = 0.85) indicated a strong initial agreement between the two raters [23,24]. Regarding quality assessments within individual studies, the kappa coefficient (κ = 0.91) indicated a strong initial agreement between the two raters [23,24]. Quality assessment of these 40 selected articles showed that 30 articles were of strong quality, 8 articles were of moderate quality, and 2 articles were of weak quality (see Table 1).

## 3. Results

### 3.1. Mental Load and Mental Fatigue Assessment Instruments for Subjective Derivations

Table 2 shows the studies (*n* = 31) that have used and tested instruments for the subjective derivation of mental load and fatigue. Of these studies, 75% focused on subjective derivation. However, we observed that most of these instruments focused on terms related to mental load and fatigue but not on these specific terms. These related terms are, for example, job-related stress (e.g., Mental Workload Instrument or Fatigue Assessment Scale for Construction Workers), or chronic fatigue syndrome. Concerning the variables and instruments used to validate these instruments, most of these studies used other related scales such as the Ratio of Perceived Exertion to compare the results obtained. Some authors used the behavioral consequences of mental fatigue, such as sleep (i.e., PSQI), in comparison with the CFS and biological parameters (i.e., ECG). The population used to validate these instruments ranged from school and university students to workers and clinical patients.

### 3.2. Mental Load and Fatigue Assessment Instruments for Behavioral Derivations

Table 3 presents five studies that used instruments to analyze the behavioral derivations of mental load and fatigue. Of these works, 12.5% focused on these derivations. Cognitive functioning, using attention, eye movement, accuracy, performance drive, or reaction time, was analyzed in this type of derivation of mental fatigue. To validate these instruments for the analysis of the behavioral derivations of mental fatigue, they were compared both with scales and questionnaires (e.g., MFS or CFS), other behavioral consequences (i.e., sleep), and physiological derivations (i.e., EEG). Most of these studies were performed with healthy participants, university students, or clinical patients, showing a higher variety of population than in the validation of the previously analyzed subjective scales.

### 3.3. Mental Load and Fatigue Assessment Instruments for Physiological Derivations

Finally, Table 4 presents a total of five studies that designed a battery of tests or instruments to analyze the physiological derivations of mental fatigue. Of these studies, 12.5% focused on such derivations. The main instrument used for these derivations was the EEG. Concerning the comparison of instruments, some of these investigations used behavioral responses to compare the instruments, for example, a comparison of VAS and EEG. Samples of workers and healthy patients were used in these studies.

## 4. Discussion

The aim of the present study was to summarize the different mental load and fatigue assessment instruments used, as well as to show their accuracy, reliability, and validity according to the derivation of mental load or fatigue analyzed by these instruments. The main results showed that there is a prevalence of subjective scales to measure mental load and fatigue. However, the use of electroencephalograms appears as an emergent form to understand the biological mechanisms of mental load and fatigue.

### 4.1. Mental Load and Mental Fatigue Assessment Instruments for Subjective Derivations

Our results showed that 75% of the instruments included in the present study focused on the subjective derivations of mental load and fatigue. These results indicated a tendency to use self-reported questionnaires or scales in the analysis of mental load and fatigue. The extended use of these types of instruments may be explained by the high validity and usefulness of their measurements [63]. However, experts should take into account the context involved to choose the most valid instrument, according to the data to be extracted. Previously, Russell et al. [8] defined the complex nature of human factors, which could explain why, when analyzing mental fatigue, experts also analyzed other psychological factors. Indeed, work settings and hospitals were the main contexts where these instruments have been used, whereas in other contexts, such as schools or sports, where mental fatigue is present [64,65], few papers have analyzed the validity and reliability of these instruments [7]. As mentioned, these types of instruments are useful in the research of students and athletes because these populations usually have little time to answer our research questions [1]. The main interest of these instruments is the individualization of the feelings of mental fatigue [8]. Such individualization of the context is important from a clinical and practical viewpoint. For example, in a sports context, one task may significantly increase the mental fatigue of a certain athlete, while this same task will not change the mental fatigue of another athlete. This may be extended to hospital patients, students, or workers because mental fatigue has a subjective derivation, among others. Indeed, this situation justifies the use of these scales. However, although these types of instruments have highlighted the role of mental fatigue and promoted the study of this variable, a great number of experts have declared that further analysis of the physiological mechanisms is needed to explain mental load and fatigue [1].

### 4.2. Mental Load and Fatigue Assessment Instruments for Behavioral Derivations

Our results show that 12.5% of the instruments included in the present study focused on the behavioral derivations of mental load and fatigue. These variables allow experts to determine how mental fatigue may influence performance indicators in each context. Russell et al. [66] asked an athletic population about their symptoms in the presence of mental fatigue. These athletes felt slower, with poor reaction times and decreased accuracy. Moreover, a great number of papers have demonstrated the relationship between an increase in the feelings of mental fatigue and a decrease in the specific behavioral performance in different areas [34,52,67]. On the contrary, the results of the present study suggest that few instruments have been validated for this purpose from a behavioral perspective. From a clinical and practical point of view, this implies a limitation in the analysis of the negative effects of mental fatigue. Mental fatigue is important because of its negative consequences in surgeons, athletes, or performance and health drivers. More studies designing instruments for behavioral derivations or examining the effects of mental fatigue in human behavior are necessary to further analyze the importance of mental fatigue.

### 4.3. Mental Load and Fatigue Assessment Instruments for Physiological Derivations

Finally, our results show that 12.5% of the instruments included in the present study focused on the physiological derivations of mental load and fatigue. The influence of the brain in mental fatigue has been demonstrated; indeed, this influence has allowed researchers to differentiate the mental and physical nature of fatigue [2]. Whereas physical fatigue is normally caused by an impairment in the traditional physiological systems, such as heartrate or blood lactate, impairments in these systems have not been observed in the performance-related decreases in mental fatigue [2]. This shows that less is known about the psychobiological processes involved in mental fatigue. Although the complexity of these instruments (price, complexity, time…) could explain the few papers published about these instruments, this information would allow researchers to understand the mechanisms that underly the presence of mental fatigue and its consequences [7]. This information is interesting from a clinical and practical viewpoint. For example, it would be useful to know how mental fatigue can be manipulated, how recovery strategies can be used, or how to maintain performance in presence of mental fatigue. Indeed, as mentioned, a large number of experts support the importance of further analysis of this derivation to advance in this topic.

## 5. Strengths and Limitations

This investigation presents a series of noteworthy strengths. Firstly, to our knowledge, no previous studies have studied the instrument used to analyze mental load and fatigue. Indeed, no previous studies have classified the instruments used to analyze mental fatigue according to the type of derivation.

The present research also presents some limitations that should be mentioned. The main limitation of this systematic review is the difficulty to obtain definitive conclusions, based on the heterogeneity of the type of instruments (e.g., scales, EEG, questionnaires).

## 6. Practical Applications and Future Research

The main practical application of this investigation is that these data could allow experts to choose an adequate instrument to analyze mental fatigue according to their needs. Experts could even design a battery of instruments to analyze mental fatigue from a global perspective.

For future research, we highlight the need to design specific instruments to quantify mental load and fatigue in sports or education. In addition, the use of behavioral and objective measures (e.g., blood sample or EEG) would allow further analysis of the causes and consequences of mental load and fatigue.

## 7. Conclusions

Of the studies included, 76% focused on the subjective derivation of mental fatigue. Therefore, we can conclude that most of the existing instruments to analyze mental load and fatigue are subjective questionnaires and scales. Furthermore, 12.5% analyzed the behavioral derivation, and 12.5% analyzed the physiological derivation of mental load and fatigue. Thus, few studies have designed instruments to quantify these variables from behavioral and physiological derivations. The scales have allowed experts to highlight the role of mental fatigue, which is important to assess the individual effect of mental load and fatigue in each subject. However, experts also stress the need to study the mechanisms involved in mental load and fatigue, analyzing the physiological mechanisms. More information is also necessary for sports and schools to analyze mental fatigue because most of these works were carried out with hospital patients and workers.

## Figures and Tables

**Table 1:** Quality assessment ‘Qualsyst’.

Study	A	B	C	D	E	F	G	H	I	J	K	L	M	N	Quality Score	Quality Classification
Chilcot et al. (2016) [27]	2	1	2	1	N/A	N/A	N/A	2	1	2	2	N/A	2	2	0.77	Strong
Cho et al. (2007) [28]	2	2	2	2	N/A	N/A	N/A	2	2	1	1	N/A	2	1	0.77	Strong
Chiu et al. (2018) [29]	2	2	2	2	N/A	N/A	N/A	2	1	2	1	2	2	2	0.91	Strong
Duan and Mu (2018) [30]	2	2	2	2	0	N/A	0	2	2	2	2	N/A	2	2	0.83	Strong
Fong et al. (2015) [31]	2	2	2	2	1	N/A	N/A	2	2	2	2	N/A	2	2	0.95	Strong
Visser-Keizer et al. (2015) [32]	2	2	N/A	2	N/A	N/A	N/A	2	N/A	2	2	N/A	2	2	1	Strong
Friedrich et al. (2018) [33]	2	2	2	2	N/A	N/A	N/A	2	2	2	2	N/A	2	2	1	Strong
Knobel et al. (2003) [34]	2	2	2	2	2	N/A	N/A	2	2	2	2	2	2	2	1	Strong
Porro et al. (2019) [35]	2	2	1	1	N/A	N/A	N/A	2	1	2	2	N/A	2	2	0.85	Strong
Zhang et al. (2015) [36]	2	2	1	2	N/A	N/A	0	0	1	2	N/A	N/A	1	2	0.65	Moderate
Kauffman et al. (2019) [37]	2	2	2	2	N/A	N/A	N/A	2	2	2	2	N/A	2	2	1	Strong
Kumamoto and Arai (2004) [38]	2	2	1	1	N/A	N/A	N/A	2	1	1	2	N/A	1	2	0.75	Strong
Dębska et al. (2013) [39]	2	2	1	1	N/A	0	0	1	2	2	0	N/A	2	2	0.75	Strong
Bertram et al. (1990) [40]	2	2	1	1	N/A	N/A	N/A	2	1	2	2	N/A	2	2	0.85	Strong
Chuang et al. (2018) [41]	2	2	2	2	N/A	N/A	N/A	2	2	2	2	N/A	2	2	1	Strong
Chung et al. (2014) [42]	2	2	2	2	2	N/A	N/A	2	2	2	2	1	2	2	0.96	Strong
Elbers et al. (2012) [43]	2	2	2	2	2	N/A	N/A	2	1	2	2	1	2	2	0.92	Strong
Hagelin et al. (2007) [44]	2	2	2	1	1	N/A	N/A	2	2	2	2	1	2	1	0.83	Strong
Gentile et al. (2003) [45]	2	2	2	2	N/A	N/A	N/A	1	2	2	2	N/A	2	2	1	Strong
Munch et al. (2006) [46]	2	2	2	1	N/A	N/A	N/A	2	2	2	2	N/A	2	2	0.95	Strong
Schubart et al. (2019) [47]	2	2	2	2	N/A	N/A	N/A	2	2	2	2	N/A	2	2	1	Strong
Burke et al. (2018) [48]	2	2	2	2	N/A	N/A	N/A	2	2	2	0	N/A	2	2	0.90	Strong
Krell (2017) [12]	N/A	2	2	2	N/A	N/A	N/A	2	2	2	2	N/A	2	2	0.90	Strong
Lin and Cai, (2009) [49]	2	1	N/A	N/A	N/A	N/A	N/A	N/A	N/A	0	N/A	N/A	0	0	0.30	Weak
Yang and Wu (2005) [50]	2	2	2	2	N/A	N/A	N/A	2	2	2	N/A	N/A	2	2	0.90	Strong
Couvy-Duchesne et al. (2017) [51]	2	2	2	1	2	N/A	N/A	1	2	2	1	1	2	2	0.83	Strong
Shuman-Paretsky et al. (2017) [52]	2	2	2	2	N/A	N/A	N/A	2	2	2	2	N/A	2	2	1	Strong
Ceballos-Vásquez et al. (2016) [11]	2	1	2	2	N/A	N/A	N/A	1	2	1	0	N/A	2	2	0.75	Moderate
MeAuley and Courneya (1994) [53]	1	2	2	2	N/A	N/A	N/A	1	2	1	2	N/A	2	2	0.85	Strong
Abma et al. (2013) [54]	2	2	2	0	N/A	0	N/A	2	2	2	N/A	N/A	2	2	0.80	Strong
Cimprich et al. (2011) [55]	2	2	2	2	N/A	N/A	N/A	1	2	2	2	N/A	2	2	0.79	Strong
Di Stasi et al. (2012) [56]	2	1	1	2	0	0	0	0	0	2	0	N/A	2	2	0.46	Weak
Puspasari et al. (2017) [57]	2	2	1	1	0	0	0	2	1	2	1	N/A	2	2	0.62	Moderate
Price et al. (2017) [58]	1	2	2	2	2	0	2	2	0	2	1	N/A	2	2	0.77	Strong
Crocetta et al. (2014) [59]	2	1	2	2	N/A	N/A	N/A	2	2	2	2	N/A	2	2	0.95	Strong
Neal et al. (2014) [60]	2	2	1	2	0	0	0	2	1	2	2	N/A	2	2	0.69	Moderate
Liu et al. (2016) [61]	2	2	N/A	1	N/A	N/A	N/A	N/A	N/A	2	N/A	N/A	2	2	0.69	Moderate
Gharagozlou et al. (2015) [62]	2	2	2	1	N/A	0	0	0	1	2	0	N/A	2	2	0.58	Moderate
Patel et al. (2018) [25]	2	2	1	1	0	0	0	1	1	2	1	N/A	1	2	0.54	Moderate
Sun et al. (2014) [15]	1	N/A	N/A	2	N/A	N/A	N/A	2	N/A	2	2	N/A	2	2	0.59	Moderate

Note. Articles were presented in the same order as the Tables’ results. Criteria = 1. Alphabetical order of the instrument; 2. Alphabetical order of the authors. Punctuations: Yes = 0; partial =1, no =0. Variables: A = Question described; B = Appropiate study design; C = Appropiate subject selection; D = Characteristics described; E = Random allocation; F = Researchers blinded; G = Subjects blinded; H = Outcome measures well defined and robust againts bias ; I = Sample size appropiate; J = Analytic methods well described; K = Estimate of variance reported; L = Controlled for confounding variables; M = Results reported details; N = Conclusions reported by results.

**Table 2:** Mental load and mental fatigue assessment instruments for subjective derivations.

Mental Load or Fatigue Instrument	Authors	Sample	Instruments Used to Compare	Results	Conclusions
Chalder Fatigue Questionnaire (CFQ)	Chilcot et al. (2016)	444 participants with multiple sclerosis (*M* = 45.15, *SD* = 12.35).	CFQ Work and Social Adjustment Scale (WSAS) Multidimensional Fatigue Inventory (MFI) Hospital Anxiety and Depression Scale (HADS).	Reliability coefficients for mental and physical subscales were both 0.96.	CFQ is a valid and reliable instrument to measure fatigue severity in people with multiple sclerosis.
	Cho et al. (2007)	207 primary care patients, between 18 and 45 years old.	12-item General Health Questionnaire (GHQ-12) Revised Clinical Interview Schedule (CIS–R).	The Brazilian CFQ’s internal consistency improved slightly from the pilot study to the validation study: Cronbach’s alpha from 0.86 to 0.88.	Brazilian CFQ had good reliability and validity, which have improved during the intercultural adaptation and validation process.
Chinese Mental Fatigue Scale (CMFS)	Chiu et al. (2018)	150 traumatic brain-injured adults for 6 months (*M* = 50.90).	Clinical Useful Depression Outcome Scale Chinese version (CUDOS)	Correlations between the items and the total scores ranged from 0.48 to 0.81 for the 13-item MFS (all *p* < 0.001).	CMFS has satisfactory statistical properties to quantify mental fatigue in traumatic brain-injured patients.
Chinese version of Stress Overload Scale-Short(SOS-SC)	Duan and Mu (2018)	1364 adults (*M* = 40.00; *SD* = 7.60).	Multidimen-sional Scale of Perceived Social Sup-port Depression Anxiety Stress Scale Brief Inventory of Thriving.	Personal vulnerability and workload were positively and significantly correlated with the score of SOS-SC.	SOS-SC can be used to measure stress and mental health status in the Chinese population.
Chronic Fatigue Syndrome (CFS) Chinese Version	Chiu et al. (2018)	150 traumatic brain-injured adults for 6 months (*M* = 50.90).	CUDOS	Correlations between the items and the total scores ranged from 0.39 to 0.81 for the 14-item CMFS (*p* < 0.001)	CFS has satisfactory statistical properties to quantify mental fatigue in traumatic brain-injured patients.
	Fong et al. (2015)	1259 adults from different jobs (*M* = 43.0, *SD* = 8.0).	4-point Chinese Hospital Anxiety Depression Scale19-item Chinese Pittsburgh Sleep Quality Index (PSQI) 12-item Chinese Short-Form Health Survey.	Three factors of CFS (physical fatigue, low energy, and mental fatigue) were positively correlated with anxiety (*r* = 0.32–0.47, *p* < 0.01), depression (*r* = 0.31–0.50, *p* < 0.01), and exhaustion (*r* = 0.41–0.59, *p* < 0.01), and weakly correlated with sleep disorders (*r* = 0.21 –0.30, *p* < 0.01).	CFS is a valid measure of fatigue symptoms in the general population.
Dutch Multifactor Fatigue Scale	Visser-Keizer et al. (2015)	148 participants, 9 with stroke, 5 with traumatic brain injury, 55 with ischemic stroke, 22 with hemorrhagic stroke, 22 with acquired brain injury, and 35 with traumatic brain injury.	No	Good reliability is shown for mental fatigue (ICC > 0.80). Patients without injuries reported significantly greater mental fatigue than patients with injury.	This questionnaire diagnoses fatigue.
EORTC QLQ-FA12 quality of life questionnaire	Friedrich et al. (2018)	577 participants (*M* = 30.3, *SD* = 6.1).	EORTC QLQ-C30 questionnaire. HADS. Supportive Care Needs Short Form 34 items.	The cognitive fatigue items’ reliability ranged from 0.45 to 0.73. The correlations between the three scales ranged between 0.63 and 0.70. Cronbach’s alpha for cognitive fatigue was 0.73.	This instrument can discriminate between physical, emotional, and cognitive fatigue.
	Knobel et al. (2003)	238 advanced cancer patients and 128 cancer survivors (*M* = 52.50).	Fatigue Questionnaire (FQ)	FA scale correlated between 0.49 and 0.75 at all assessment points with the Physical Fatigue (PF) and Mental Fatigue (MF) scales of the FQ.	EORTC QLQ C30 fatigue scale meaasures fatigue as part of an overall fatigue assessment.
	Porro et al. (2019)	68 breast cancer patients (*M* = 46.97, *SD* = 6.92).	MFI-20.	Univariate analyzes showed Return To Work (RTW) probability was reduced by high scores for mental fatigue, *r* = 0.85, *p* <0.05. Only the change in mental fatigue during treatment influenced the RTW probability.	Attention should be paid to the use of validated scales to evaluate mental constructs.
Fatigue Assessment Scale for Construction Workers (FASCW).	Zhang et al. (2015)	144 unionized construction workers in New England, from 19 to 60 years (*M* = 42.4; *SD* = 10.3).	Ratio of Perceived Exertion (RPE) Profile of Mood States (POMS).	Results indicated significant high correlations between FASCW and the Fatigue subscale of POMS and the measure of RPE.	FASCW is a promising instrument for assessing a general concept of fatigue.
Functional Status Questionnaire	Kauffman et al. (2019)	1287 undergraduate students (*M* = 21.68, *SD* = 4.54).	Anxiety Sensitivity Index Inventory of Depression and Anxiety Symptoms Positive and Negative Affect Schedule (PANAS).	FSQ had excellent internal consistency (α = 0.92). Total FSQ score was positively associated with anxiety sensitivity (*r =* 0.49), general depression (*r =* 0.37), social anxiety (*r =* 0.40), panic (*r =* 0.43), and negative affectivity (*r =* 0.37).	FSQ may be a valid and promising approach to better understand the implications of fatigue in real-world contexts (e.g., primary care).
J-ZBI-8 Questionnaire	Kumamoto and Arai (2004)	315 subjects who lived with primary caregivers (*M* = 81.2, *SD* = 7.5).	No	No clear relationship between the nursing care load and nursing time was found. This relationship is significantly related to the attention load.	The J-ZBI _ 8 questionnaire has two subscales whose factorial structure is clearly defined.
Meister Questionnaire	Dębska et al. (2013)	211 nurses (*M* = 43.1, *SD* = 7.26).	Maslach Burnout Inventory.	Cronbach’s alpha was 0.83 for the total score.	Meister questionnaire meets the psychometric criteria of reliability and validity to assess mental load in nurses.
Mental WorkLoad Instrument.	Bertram et al. (1990)	48 patients admitted to clinical care, between 31 and 45 years old.	No	Significant correlations were observed between the work demand, satisfaction, and self-perceived performance.	Mental workload correlated directly and inversely with both satisfaction and the self-rated quality of the patient care provided.
MFI	Chuang et al. (2018)	123 participants (43 males and 80 females; *M* = 46.12, *SD* = 18.40).	PSQI Survey of Short Format Health (SF-36-T) questionnaire.	Results showed moderate convergent validity by correlating fatigue with quality of life, including sleep.	Results support the use of the MFI traditional Chinese version as an integral instrument to measure specific fatigue aspects.
	Chung et al. (2014)	137 major depressive disorder (MDD) patients (*M* = 49.6, *SD* = 9.6).	Scale to assess the severity of Major Depression and Associated Symptoms (HDRS) HADS, Insomnia Symptom Self-Assessment Scale (ISI) SF-36.	MFI-20 has good internal consistency (Cronbach’s alpha = 0.89). Suitable concurrent validity, significant correlations between MFI-20 scores and depressive and anxiety symptoms, general health, and quality of life.	MFI-20 is a valid and reliable instrument to assess fatigue in MDD patients with residual symptoms.
	Elbers et al. (2012)	153 patients diagnosed with Parkinson’s disease (*M* = 67.07, *SD* = 7.54).	No	All subscales showed suitable internal consistency reflected by a Cronbach range of 0.74 to 0.92.	MFI is a reliable and valid instrument to evaluate the multidimensional aspects of fatigue in Parkinson patients.
	Hagelin et al. (2007)	594 cancer patients (*M* = 59.50).	Borg Scale (CR-10).	Cronbach values in the MFI-20 ranged between 0.67 and 0.94. The correlation between the MFI-20 subscales and the CR-10 scores ranged between 0.37 and 0.74.	MFI-20 Swedish version is a valid and reliable instrument for measuring fatigue in different patient populations and in healthy individuals.
	Gentile et al. (2003)	225 participants (*M* = 52, *SD* = 15).	VAS	Correlations between each subscale and VAS are highly significant (*p* < 0.001).	MFI French version shows that this instrument is valid for clinical application.
	Munch et al. (2006)	278 advanced cancer patients (*M* = 64).	HADS.	The two psychological subscales of MFI-20, Mental Fatigue and Reduced Motivation, were significantly associated with each other. Only General Fatigue and Mental Fatigue correlated significantly with the HADS Anxiety subscale.	MFI-20 may be a useful tool for further research on fatigue etiology.
	Schubart et al. (2019)	175 patients with Ehlers-Danlos Syndromes (*M* = 42.40).	Wisconsin Brief Pain Inventory Epworth Sleepiness Scale (ESS) PSQI Beighton Score Psychological Inventory (SCL-90) Sleep Medicine Associates of Maryland	Mental fatigue was correlated with pain (*r* = 0.16), night sleep (*r* = 0.20), daily sleep (*r* = 0.35), and dysautonomia (*r* = 0.36).	This research shows the relation between mental fatigue and other constructs.
Pittsburgh Fatigability Scale (PFS).	Burke et al. (2018)	35 healthy old people (*M* = 73.77, *SD* = 5.9).	MFIS HADS PSQI ESS Montreal Cognitive Assessment (MOCA) Operation Span Task (OSPAN)	PFS mental fatigue subscores highly correlated with the EES scores (ρ = 0.63, *p* < 0.001). PFS mental fatigue scores also correlated with the MFIS cognitive score (ρ = 0.36, *p* = < 0.05).	The lack of correlation between task-based fatigability measures and the PFS Mental subscale may indicate that mental fatigue is difficult to capture using questions about fatigue based on previous or imaginary experiences.
Mental Load (ML) and Mental Effort (ME) Questionnaire of Students in Biology Education (StuMMBE-Q).	Krell (2017)	602 students (9 and 10 school grades; from 13 to 18 years old; 52% females).	No	Results suggest that StuMMBE-Q classifies students who report low, medium, and high levels of ML and ME.	Findings suggest that the questionnaire measures two theoretically established cognitive load dimensions (mental load and mental effort) well.
Rating Scale Mental Effort (RSME)	Lin and Cai (2009)	Drivers.	Electrocardiogram (ECG) Continuous Mental Workload Scale (CBC-MWL).	Correlation coefficient between RSME and ECG is 0.85. ECG and CBC-MWL measurement show a high correlation with the RSME score.	Proposed method is consistent with the RSME method but RSME cannot be completed in real time.
Situational Fatigue Scale	Yang and Wu (2005)	96 patients (*M* = 31.10, *SD* = 10.0) and 62 university students (*M* = 21.0, *SD* = 1.99).	Fatigue Assessment Instrument Mental Fatigue Subscale Physical Fatigue Subscale (PFSubscale)	Cronbach coefficients indicated good internal consistency for the global scale (0.90), as well as for the PFSubescale (0.88) and the MFS (0.89).	SFS presents a new way to measure fatigue dimension that is different from what is measured with conventional fatigue rating scales.
Somatic and Psychological Health Report.	Couvy-Duchesne et al. (2017)	5148 participants (*M* = 15.52; *SD* = 0.75).	No	Questionnaire could be reduced to 21 items.	This questionnaire could be relevant to assess anxiety, depression, and chronic fatigue.
State-Trait Inventory for Cognitive Fatigue (STI-CF).	Shuman-Paretsky et al. (2017)	175 participants, over 65 years old (*M* = 77.35, *SD* = 6.91).	Brief Fatigue Inventory Geriatric Depression Scale Trail Making Test RBANS	The 4 components (cognitive fatigue, mental effort, motivation, and boredom) had good reliability. Strong positive relationship between cognitive fatigue and a subjective measure of general fatigue (*p* < 0.001).	The STI-CF had significant relationships in the expected direction with several variables of cognitive and health outcomes.
Subjective Exercise Experiences Scale (SEES).	MeAuley and Courneya (1994)	454 university students (*M* = 20.78; *SD* = 2.18).	No	The comparison between the three scales of SEES showed their reliability: Positive Well-Being (PWB) α = 0.36, Psychological Distress (PD) α = 0.25, and Fatigue α = 0.88.	Three dimensions of the SEES provide initial support for the multidimensional measurement of the capacity of psychological response to the properties of exercise stimulus: Positive well-being, psychological distress, and fatigue.
Subjective Scale of Mental Workload (SCA)	Ceballos-Vásquez et al. (2016)	379 workers (*M* = 37.36; *SD* = 10.53) of Critical Patient Units (UPC) of three Chilean hospitals.	SUSESO-ISTAS 21 questionnaire.	There are positive and significant correlations between the global mental load scores and all the psychosocial dimensions of the SUSESO-ISTAS 21 (*p* < 0.05).	SCAM presents high reliability and suitable validity in a Chilean sample for mental load evaluation.
WRFQ	Abma et al. (2013)	553 workers between 18 and 64 years old who worked 12 h weekly.	Endicott Work Productivity Scale Physical Component Summary Short Form—12 Checklist Individual Strength Need for Recovery Subscale Job Content Questionnaire Work Ability Index Utrecht Work Engagement Scale Work Involvement Scale.	Cronbach’s alpha coefficients were calculated for each WRFQ subscale and the total score (ideal, between 0.70 and 0.95).	WRFQ 2.0 is a reliable and valid instrument to measure the health-related work functioning in the working population in general.

**Table 3:** Mental load and mental fatigue assessment instruments for behavioral derivations.

Mental Load or Fatigue Instrument	Authors	Sample	Instruments Used to Compare	Results	Conclusions
Attentional Function Index (AFI)	Cimprich et al. (2011)	172 women diagnosed with breast cancer. Ages between 27 and 86 years old.	No	Internal consistency coefficient (Cronbach’s α) for the revised 13-item scale was 0.92, indicating satisfactory reliability.	Findings indicate that AFI is a valid and reliable measure to assess the perceived detrimental effects of cognitive dysfunction in chronic and life-threatening diseases, such as breast cancer.
Eyelink 1000 Remote Eye Tracking System	Di Stasi et al. (2012)	10 healthy volunteers. Five women and five men (*M* = 23.9, *SD* = 4.9).	SIRCA Simulator Groningen Sleep Quality Scale Stanford Sleepiness Scale CFS Mental Workload Test	The peak velocity of saccadic eye movements decreased after driving (*p* < 0.05), due to mental fatigue.	Saccadic eye parameters, particularly the peak velocity, are a sensitive indicator of mental fatigue.
Logitech Driving Simulator with Citycar Driving software	Puspasari et al. (2017)	Seven commercial drivers, between 25 and 35 years old.	Electroencephalogram (EEG) Karolinska Sleepiness Scale	All measured parameters showed significant changes related to driving duration (*p* < 0.05).	Results show alpha, beta, theta, and delta bands are significantly different before and after driving, with an increase in the theta-delta band and a decrease in the alpha-beta band. These correlate with poor driving performance.
Psychomotor Vigilance Test (PVT)	Price et al. (2017)	21 participants (*M* = 22, *SD* = 4).	Mental Arithmetic Test Spatial Span Test MFS	Only the mobile test PVT is valid and reliable to assess cognitive accuracy. The arithmetic test does not show a strong correlation with MFS.	The mobile application is considered a potentially effective tool for the individual assessment of cognitive fatigue levels More continuity in time is needed and the test must be carried out daily.
TRT_S software	Crocetta et al. (2014)	216 university students, between 17 and 45 years old (*M* = 24, *SD* = 6).	Vienna Test System (VTS)	Intraclass coefficient correlation of TRT in young adults showed a strong correlation between Simple TRT and VTS (*r* = 0.72).	Results confirmed the TRT_S 2012 software’s validity, as a reliable cognitive test to assess the influence of mental fatigue on cognitive performance.

**Table 4:** Mental load and mental fatigue assessment instruments for objective derivations.

Mental Load or Fatigue Instrument	Authors	Sample	Instruments Used to Compare	Results	Conclusions
Air Traffic Workload Input Technique	Neal et al. (2014)	16 licensed air traffic controllers.	Task Load Metrics.	The model explained 42% of the variance in workload after controlling for differences among raters.	The final model provided a reasonable fit to the data, despite including only five predictors. It can thus be considered a multilevel unified dynamic density model.
Cognitive Pilot-Aircraft Interface (CPAI) procedures.	Liu et al. (2016)	Airplane pilots.	No	Higher heart rates are related to higher fatigue levels and the flickering speed demonstrates a similar relationship. For mental fatigue, the heart rate is more important than the blink rate.	Simulation results demonstrate a preliminary validity of CPAI system for this purpose. Estimated human cognitive states are consistent both with external conditions and physiological states.
EEG	Gharagozlou et al. (2015)	12 healthy male drivers (*M* = 23.8, *SD* =1.44; from 20 to 30 years old). Subjects had a valid driver’s license with at least 2 years driving experience and had no brain injuries history.	VAS.	Significant increase in absolute alpha power (*p* = 0.006), as well as in F-VAS scores were observed during the final driving section (*p* = 0.001).	The study suggested that variations in alpha power could be a good indicator of drivers’ mental fatigue.
	Patel et al. (2018)	18 participants of different jobs	ECG.	The use of EEG spectral power in all bands obtains better performance for mental fatigue assessment (*p* < 0.001).	The use of EEG spectral power characteristics across the entire range of physiological bands allows a better representation of all mental states.
	Sun et al. (2014)	26 right-handed and neurologically normal participants (*M* = 22.20; *SD* = 1.53).	No	Few functional connections were significantly associated with mental fatigue (*p* > 0.05).	Viability demonstration of a method of assessing mental fatigue based on functional connectivity.

## Data Availability

Not applicable.
